# Deviations From the Ideal Plasma Volume and Isolated Tricuspid Valve Surgery—Paving the Way for New Risk Stratification Parameters

**DOI:** 10.3389/fcvm.2022.849972

**Published:** 2022-03-25

**Authors:** Ena Hasimbegovic, Marco Russo, Martin Andreas, Paul Werner, Iuliana Coti, Dominik Wiedemann, Alfred Kocher, Günther Laufer, Benedikt S. Hofer, Markus Mach

**Affiliations:** ^1^Division of Cardiology, Department of Internal Medicine II, Medical University of Vienna, Vienna, Austria; ^2^Division of Cardiac Surgery, Department of Surgery, Medical University of Vienna, Vienna, Austria; ^3^Division of Gastroenterology and Hepatology, Department of Internal Medicine III, Medical University of Vienna, Vienna, Austria

**Keywords:** congestion, plasma volume status, tricuspid valve, isolated tricuspid valve surgery, mortality

## Abstract

**Background:**

Congestion and plasma volume expansion are important features of heart failure, whose prognostic significance has been investigated in a range of surgical and non-surgical settings. The aim of this study was to evaluate the value of the estimated plasma volume status (ePVS) in patients undergoing isolated tricuspid valve surgery.

**Methods:**

This study included patients who underwent isolated tricuspid valve surgery at the Vienna General Hospital (Austria) between July 2008 and November 2018. The PVS cut-off was calculated using ROC analysis and Youden's Index.

**Results:**

Eighty eight patients (median age: 58 [IQR: 35-70] years; 44.3% male; 75.6% NYHA III/IV; median EuroSCORE II 2.65 [IQR: 1.70-5.10]; 33.0% endocarditis-related regurgitation; 60.2% isolated repair; 39.8% isolated replacement) were included in this study. Patients who died within 1 year following surgery had significantly higher baseline ePVS values than survivors (median ePVS 5.29 [IQR: −1.55-13.55] vs. −3.68 [IQR: −10.92-4.22]; *p* = 0.005). During a median actuarial follow-up of 3.02 (IQR: 0.36-6.80) years, patients with a preoperative ePVS ≥ −4.17 had a significantly increased mortality (log-rank *p* = 0.006).

**Conclusions:**

ePVS is an easily obtainable risk parameter for patients undergoing isolated tricuspid valve surgery capable of predicting mid- and long-term outcomes after isolated tricuspid valve surgery.

## Introduction

The prevalence of heart failure and acute decompensated heart failure in patients undergoing isolated tricuspid valve surgery is high, and its presence is associated with poor outcomes (1–3). Isolated tricuspid valve surgery is a comparatively rare procedure with a high mortality (1, 4–9). Due to the low overall number of isolated tricuspid valve surgeries performed yearly, specific risk stratification parameters have not been explored as thoroughly as those in other types of cardiac surgery. Similarly, the relevance of noninvasive laboratory indicators of congestion has not been assessed in this patient collective. In this paper, we attempted to evaluate one such index, the estimated plasma volume status, for the risk stratification of patients undergoing isolated tricuspid valve surgery.

Congestion and volume overload, the hallmark features of heart failure, affect both the interstitial and the plasma space and develop gradually, with an initially asymptomatic clinical course. However, ultimately the majority of patients presenting with decompensated heart failure exhibit clinical signs of congestion (10, 11). The degree to which diuretic therapy affects congestion differs between the interstitial and intravascular compartments, which limits the usefulness of a mere weight-based assessment for tracking the success of in-hospital volume management of decompensated patients with heart failure (12, 13).

Although methods for assessing the degree of intravascular congestion via pressure measurement in the right atrium or the assessment of the pulmonary capillary wedge pressure are available, their invasiveness limits their applicability in day-to-day practice and they are thus mainly reserved for specific high-risk settings (10). Surrogate markers for the assessment of the intravascular volume overload, such as the brain natriuretic peptide (BNP), secreted in response to mechanical stretching of the cardiomyocytes in a volume overloaded left ventricle, have long been used to diagnose, assess the severity of and guide treatment of heart failure (14). The current gold standard for the direct measurement of the plasma volume is the nuclear medicine blood volume assessment. This set of methods is based on the injection of a tracker substance, followed by blood sampling to determine its dilution and subsequently extrapolate the dilution volume. Such dilution-based methods, albeit with the use of rudimentary dyes, were conducted as early as one century ago, but the time-consuming nature, the required expertise and their susceptibility to measurement errors have impeded their wider implementation (15–17). However, recent technical advances have resolved some of these issues and might make such techniques more useful in the emergency and other settings in the future (18).

Multiple methods use the patient's weight, hematocrit or hemoglobin for the non-invasive assessment of the plasma volume status (PVS), such as the Strauss, Duarte and Hakim formula (19–21). The correlation of these calculated estimates with symptoms and diagnostic markers of congestion has not been sufficiently elucidated, although initial findings have proposed some correlations with imaging modalities (22). In recent years, several studies have found compelling evidence for the prognostic value of the estimated PVS in cardiovascular disease. Duarte et al. found that PVS can predict the likelihood of early cardiovascular events following acute myocardial infarction with acute heart failure (20). Martens et al. found that PVS correlates with the measured plasma volume assessed by technetium red blood cell labeling and predicts overall mortality and heart-failure related hospitalization in a large cohort of patients with different etiologies of heart failure (23). Kobayashi et al. undertook an extensive assessment of Duarte's PVS in heart failure with a preserved ejection fraction (HFpEF) and found that it had a high prognostic value for adverse events, accurately reflected the degree of congestion, was not significantly impacted by renal function and could enhance existing risk stratification accuracy in conjunction with other established parameters (24). Similarly promising results for the use of PVS for risk stratification of HFpEF patients were also described by Huang et al. and Grodin et al. (25, 26). Tamaki et al. followed a group of patients admitted for acute decompensated heart failure and found that the plasma volume status correlated with overall mortality and rehospitalization for decompensated heart failure, findings similar to those of Yoshihisa et al. (27, 28). The value of PVS for risk stratification has been examined for a range of other applications, including left ventricular assist device (LVAD) recipients, coronary artery bypass grafting (CABG) or acute respiratory distress syndrome (ARDS) (29–31). Several studies have also looked at the potential of PVS for outcome and mortality prediction in patients undergoing interventions for valvular disease. A large-scale study of patients who underwent transcatheter aortic valve replacement (TAVR) by Shimura et al. found a link between a combined PVS and NYHA class stratification and mortality, as well as heart-failure-related rehospitalization (32). The association of PVS with outcomes following TAVR was also demonstrated by Seoudy et al. (33). A study by Schaefer et al. examined the association between PVS and mortality following mitral valve surgery (34).

However, the possible link between the calculated PVS and mortality following tricuspid valve surgery has not yet been explored. Thus, with this study we aimed to examine a possible link between the calculated PVS as a surrogate marker of congestion and the survival of patients undergoing isolated tricuspid valve surgery.

## Methods

### Patient Selection and Preoperative Evaluation

For this study, data from 88 consecutive patients who underwent isolated tricuspid valve surgery at the Department of Cardiac Surgery, Medical University of Vienna between July 2008 and November 2018 was retrospectively analyzed. The isolated tricuspid valve surgery was conducted according to standard institutional operating procedure and the internal guidelines of the Division of Cardiac Surgery, Medical University of Vienna.

The preoperative patient assessment included a measurement of body weight, height, and standard laboratory tests. The etiology and degree of tricuspid valve regurgitation or tricuspid valve disease, as well as the presence of active endocarditis were recorded. A detailed patient history including comorbidities, risk factors, substance abuse and previous cardiac surgeries was collected. In accordance with routine practice, the EuroSCORE II and NYHA class were assessed.

This study was approved by the Ethics Committee of the Medical University of Vienna and was conducted in accordance with the 1964 Declaration of Helsinki, as well as its later amendments, and did not receive any funding (EK: 1289/2019).

### Plasma Volume Equations

The preoperative PVS values were calculated according to two separate formulae using the patient's weight, hemoglobin, and hematocrit.

First, the actual plasma volume (aPV) was calculated using the Hakim formula (19):


$$\begin{array}{l} aPV=(1-\;hematocrit)\times \;[a+(b\;\text{× }weight)] \end{array}$$

where hematocrit is given as a fraction and the weight refers to the weight in kilograms. The coefficient *a* has a value of 864 for female and 1,530 for male patients and the coefficient *b* has a value of 47.9 for female and 41.0 for male patients.

The ideal plasma volume (iPV) was calculated according to the equation previously published by Longo et al. (35):


$$\begin{array}{l} iPV=c\times weight \end{array}$$

where the weight is stated in kilograms and the coefficient c has a value of 40 for women and 39 for men. The PVS was calculated from the two abovementioned values using the following formula:


$$\begin{array}{l} ePVS=\frac{aPV-iPV}{iPV}\;\times 100 \end{array}$$

The second method used to assess the plasma volume status was the Duarte formula (20):


$$\begin{array}{l} Duarte^{\prime }s\;PVS=\frac{100-hematocrit}{hemoglobin} \end{array}$$

where the hemoglobin is stated in g/dl and the hematocrit is given in percentages.

### Follow-Up

All adverse events following the surgery and prior to the patient discharge were internally documented. During the observational period, all visits to the outpatient clinics and inpatient stays from the electronic health record were recorded and analyzed. Information regarding survival of patients was either available within our electronic health record or was separately obtained from the national statistics department (Statistik Austria). The last date of follow-up was recorded as either death or the last documented living visit available within our electronic records. The primary endpoint of this study was the all-cause mortality.

### Statistical Analysis

Statistical analyses were performed using the IBM SPSS 27.0 statistics software (SPSS Inc., Armonk, NY, USA) and the figures were created using GraphPad Prism V.8 (GraphPad Software, La Jolla, CA, USA). Continuous variables were reported as either the mean and standard deviation or median and interquartile range depending on their distribution pattern. The presence of a parametric distribution of metric variables was assessed using the Shapiro-Wilk test and visual analysis of the distribution. Categorical variables were recorded as the number of features with the corresponding percentage of patients. Categorical variables were compared using the Chi-square test or the Fisher's exact test. Group comparisons were conducted using either the student's *t*-test or the Mann-Whitney *U*-test for normally and non-normally distributed variables, respectively. The correlation between two metric variables was assessed via Spearman correlation coefficients. Survival analyses were performed via the Kaplan-Meier method and log-rank tests. Variables associated with a decreased survival during the follow-up period were identified using univariate and multivariate Cox regression models. Univariate *p*-values below 0.1 in the univariate analysis were considered for multivariate analysis. Variables not available for more than 5% of patients were not included in the multivariate analysis. Variables featured in the EuroSCORE II, or variables used for the calculation of or directly related to ePVS or Duarte's PVS were excluded from the multivariate analysis. In the multivariate analysis, a stepwise backward approach was applied. The accuracy of PVS in predicting the primary endpoint was examined using ROC-curve analysis based on the prediction of the 1-year mortality. The Youden Index was used to identify an ePVS of −4.17 as the optimal cut-off point. As a secondary analysis, the same procedure was applied to determine the optimal cut-off point for Duarte's PVS, which was found to be 4.79. Subsequently, the ePVS cut-off was used to stratify the patients into two groups (Group 1: ePVS < −4.17 vs. Group 2: ePVS ≥ −4.17). Two-sided *p*-values of <0.05 were considered statistically significant across the analysis.

## Results

### Baseline Clinical Characteristics and Laboratory Parameters

A total of 88 patients undergoing isolated tricuspid valve surgery were included in this study. The median age of the patients was 58 years (IQR: 35-70 years), with a median follow-up time of 3.02 (IQR: 0.36-6.80) years. 38 patients (43.2%) had functional tricuspid regurgitation at baseline, whereas one fourth (25.0%) of all patients had active endocarditis at baseline. 19 patients (21.6%) had previously undergone a surgical left heart intervention and 23 patients (26.1%) had a lead passing through the tricuspid valve. One fifth of all patients (*n* = 18 [20.5%]) either had a previous history of intravenous drug use or were active intravenous drug users at baseline. Most patients had NYHA class III/IV (*n* = 65 [75.6%]). A comprehensive overview of the baseline clinical characteristics and risk factors is provided in Table 1.

**Table 1:** Baseline characteristics of the overall and stratified cohort.

**Baseline characteristics**	**Overall cohort *n* = 88**	**Group 1 (ePVS < −4.17) *n* = 38**	**Group 0 (ePVS ≥−4.17) *n* = 50**	***P*-value**
Age (years)	58(35-70)	58(44-70)	59(33-70)	0.730
Gender (male)	39 (44.3%)	16 (42.1%)	23 (46.0%)	0.716
Height (cm)	170.0 (161-177)	180 ± 9	170 ± 10	0.830
Weight (kg)	71 (60-85)	80 (62-88)	66 (60-83)	0.074
BMI (kg/m2)	25.3 ± 4.5	26.9 (23.3-29.3)	23.5 (21.4-27.5)	0.038
LVEF (%)	60 (50-60)	60(46-60)	60 (50-60)	0.909
Previous anti-RAAS therapy	25 (28.4%)	12 (31.6%)	13 (26.0%)	0.565
Previous therapy with beta blockers	16 (18.2%)	11 (28.9%)	5 (10.0%)	0.028
sPAP (mmHg)	43(37-75)	43(34-50)	47(37-56)	0.294
Functional tricuspid regurgitation	38 (43.2%)	23 (60.5%)	15 (30.0%)	0.004
Endocarditis-related tricuspid regurgitation	29 (33.0%)	7 (18.4%)	22 (44.0%)	0.011
Tricuspid regurgitation etiology left-side related	15 (19.2%)	6 (18.2%)	9 (20.0%)	1.000
Active endocarditis	22 (25.0%)	3 (7.9%)	19 (38.0%)	0.001
Previous surgical left heart intervention	19 (21.6%)	9 (23.6%)	10 (20.0%)	0.677
Pacemaker/ICD	23 (26.1%)	6 (15.8%)	17 (34.0%)	0.054
Lead through the tricuspid valve	23 (26.1%)	6 (15.8%)	17 (34.0%)	0.054
Hemodynamic instability prior to surgery	4 (4.5%)	0 (0%)	4 (8.0%)	0.130
Any previous intravenous drug use	18 (20.5%)	3 (7.9%)	15 (30.0%)	0.015
Active intravenous drug use	7 (8.0%)	0 (0.0%)	7 (14.0%)	0.018
Any chronic hepatic condition	22 (25.0%)	4 (10.5%)	18 (36.0%)	0.007
Active hepatitis C viral infection	15 (17.0%)	3 (8.6%)	12 (24.0%)	0.084
EuroSCORE II	2.65 (1.70-5.10)	1.91 (1.22-3.71)	3.17 (2.10-6.65)	0.004
Active smoker	13 (14.8%)	4 (10.5%)	9 (18.0%)	0.379
Any previous regular smoking habit	23 (26.1%)	9 (23.7%)	14 (28.0%)	0.648
NYHA class III/IV	65 (75.6%)	24 (64.9%)	41 (83.7%)	0.044
Arterial hypertension	48 (54.5%)	19 (50.0%)	29 (58.0%)	0.455
Diabetes	12 (13.6%)	4 (10.5%)	8 (16.0%)	0.542
Previous myocardial infarction	4 (4.5%)	0 (0%)	4 (8.0%)	0.130
Previous stroke	7 (8.0%)	4 (10.5%)	3 (6.0%)	0.459
Chronic obstructive pulmonary disease	25 (28.4%)	11 (28.9%)	14 (28.0%)	0.922
Chronic kidney disease (GFR <6 ml/min or renal replacement therapy)	33 (37.5%)	10 (26.3%)	23 (46.0%)	0.059
Ongoing dialysis	5 (5.7%)	1 (2.6%)	4 (8.0%)	0.384
Extracardiac arteriopathy	3 (3.4%)	0 (38.0%)	3 (6.0%)	0.255
Carotid disease	5 (5.7%)	3 (7.9%)	2 (4.0%)	0.648
eGFR (ml/min/1.73 m2)	68.8 (49.0-102.3)	81.2 (52.4-101.1)	63.1 (45.4-105.8)	0.209
Hematocrit (%)	35.9 ± 6.6	41.8 ± 3.2	31.5 ± 4.7	<0.001
Hemoglobin (g/dl)	11.9 ± 2.3	14.0 ± 1.4	10.4 ± 1.6	<0.001
Platelet count (G/l)	200 (155-275)	193 (164-246)	206 (118-287)	0.506
Erythrocyte count (T/l)	4.1 ± 0.8	4.7 ± 0.5	3.6 ± 0.6	<0.001
MCH (pg)	29.6 (27.5-31.1)	30.1 (28.4-31.4)	29.1 (27.3-30.9)	0.212
MCV (fl)	88.4 ± 6.0	89.2 ± 5.3	87.9 ± 6.5	0.304
proBNP (pg/ml)	1,147 (433-1882)	924 (474-1672)	1,480 (400-2,351)	0.183
Creatinine (mg/dl)	1.02 (0.86-1.35)	1,03 (0.91-1.33)	1.01 (0.85-1.40)	0.584
Creatinine kinase (U/l)	48 (31-95)	72 (41-116)	40 (19-65)	<0.001
Alkaline phosphatase (U/l)	112 (79-146)	103 (72-125)	127 (91-172)	0.006
ASAT (U/l)	29 (21-41)	32 (22-43)	26 (19-38)	0.156
ALAT (U/l)	23 (17-33)	28 (21-36)	19 (12-28)	0.002
GGT (U/l)	110 (65-178)	109 (51-154)	122 (76-194)	0.259
LDH (U/l)	227 (193-303)	224 (199-289)	229 (174-304)	0.629
CRP (mg/dl)	0.63 (0.20-2.28)	0.33 (0.19-0.81)	1.29 (0.34-7.69)	<0.001
Total bilirubin (mg/dl)	0.87 (0.60-1.23)	0.87 (0.62-1.22)	0.86 (0.58-1.23)	0.860
Albumin (g/l)	40.6 (32.3-44.6)	43.6 (4.8)	34,4(9,0)	<0.001
Total protein (g/l)	73.0 (68.1-78.9)	76.1 (70.8-79.1)	71.2 (64.1-78.7)	0.033

*Data is presented as number n (%), mean ± standard deviation (SD) or median (interquartile range). BMI, body mass index; LVEF, left ventricular ejection fraction; RAAS, renin-angiotensin-aldosterone system; sPAP, systolic pulmonary artery pressure; ICD, implantable cardioverter-defibrillator; NYHA, New York Heart Association; GFR, glomerular filtration rate; eGFR, estimated glomerular filtration rate; MCH, mean corpuscular hemoglobin concentration; MCV, mean corpuscular volume; BNP, brain natriuretic peptide; ASAT, aspartate aminotransferase; ALAT, alanine aminotransferase; GGT, gamma-glutamyl transferase; LDH, lactate dehydrogenase; CRP, C-reactive protein*.

Patients with an ePVS < −4.17 had received prior therapy with beta blockers more frequently (*p* = 0.028), suffered from functional tricuspid regurgitation more frequently than the cohort with an ePVS ≥ −4.17 (*p* = 0.004), exhibited fewer cases of endocarditis-related tricuspid regurgitation (*p* = 0.011), and had a lower prevalence of previous or active intravenous drug use (*p* = 0.015 and *p* = 0.018, respectively). The ePVS < −4.17 cohort had a lower EuroSCORE II (*p* = 0.004), a higher baseline hematocrit (*p* < 0.001) and hemoglobin (*p* < 0.001), erythrocyte count (*p* < 0.001) and higher levels of serum albumin (*p* < 0.001).

### Surgical Characteristics and Adverse Events

The procedural characteristics and incidence of adverse postoperative events are shown in Table 2. Patients with an ePVS < −4.17 underwent elective surgery significantly more often (*p* < 0.001) and urgent surgery significantly less often (*p* = 0.002). These patients also underwent tricuspid repair more often and tricuspid replacement less often than the ePVS ≥ −4.17 group (*p* = 0.002). In terms of postoperative adverse events, the only significant difference between the groups was observed in the need for postoperative blood transfusion, which was required less often in the ePVS < −4.17 group (*p* = 0.003).

**Table 2:** Procedural characteristics and adverse events.

	**Overall cohort *n* = 88**	**Group 1 (ePVS < −4.17) *n* = 38**	**Group 0 (ePVS ≥ −4.17) *n* = 50**	***P*-value**
Redo surgery	31 (35.2%)	14 (36.8%)	17 (34.0%)	0.782
Elective surgery	54 (61.4%)	32 (84.2%)	22 (44.0%)	<0.001
Urgent surgery	30 (34.1%)	6 (15.8%)	24 (48.0%)	0.002
Emergency surgery	4 (4.5%)	0 (0%)	4 (8.0%)	0.130
Cardiopulmonary bypass time (min)	114 (77-140)	127 (92-159)	93 (75-129)	0.041
Isolated tricuspid repair	53 (60.2%)	30 (78.9%)	23 (46.0%)	0.002
Ring annuloplasty	49 (55.7%)	30 (78.9%)	19 (38.0%)	<0.001
Isolated tricuspid replacement	35 (39.8%)	8 (21.1%)	27 (54.0%)	0.002
Sternotomy	77 (92.8%)	30 (88.2%)	47 (95.9%)	0.184
Mini-thoracotomy	6 (7.2%)	4 (11.8%)	2 (4.1%)	0.221
Beating heart surgery	28 (31.8%)	9 (23.7%)	19 (38.0%)	0.153
Blood transfusion required	55 (62.5%)	17 (44.8%)	38 (76.0%)	0.003
Erythrocyte concentrate (units)	1.0 (0.0-3.0)	0.0 (0.0-1.3)	2.0 (1.0-4.3)	<0.001
Postoperative acute kidney injury	6 (6.8%)	1 (2.6%)	5 (10.0%)	0.229
Postoperative renal replacement therapy	4 (4.5%)	1 (2.6%)	3 (6.0%)	0.631
Postoperative pericardial effusion	5 (5.7%)	2 (5.2%)	3 (6.0%)	1.000
Postoperative ECMO support	3 (3.4%)	1 (2.6%)	2 (4.0%)	1.000
New onset atrial fibrillation	9 (10.2%)	4 (10.5%)	5 (10.0%)	1.000
Postoperative pneumonia	3 (3.4%)	1 (2.6%)	2 (4.0%)	1.000
Postoperative stroke	0 (0.0%)	0 (0.0%)	0 (0.0%)	/
Postoperative myocardial infarction	0 (0.0%)	0 (0.0%)	0 (0.0%)	/
Wound complication	7 (8.0%)	2 (5.2%)	5 (10.0%)	0.694
Pulmonary embolism	1 (1.1%)	1 (2.6%)	0 (0%)	0.432
New pacemaker implantation	9 (10.2%)	4 (10.5%)	5 (10.0%)	1.000
Postoperative stay (days)	15(9-23)	12(8-19)	17(10-34)	0.039
Re-exploration for bleeding	12 (13.6%)	4 (10.5%)	8 (16.0%)	0.542
In-hospital death	7 (8.0%)	2 (5.2%)	5 (10.0%)	0.694
1-year death	18 (20.5%)	2 (5.2%)	16 (32.0%)	0.003
Death on last follow-up	34 (38.6%)	9 (23.7%)	30 (60.0%)	<0.001

*Data is presented as number n (%), mean ± standard deviation (SD) or median (interquartile range). ECMO, extracorporeal membrane oxygenation*.

### ePVS and Duarte's PVS in Survivors and Non-survivors

The difference in ePVS and Duarte's PVS in survivors and non-survivors at two timepoints during follow-up is shown in Table 3.

**Table 3:** ePVS and Duarte's PVS in survivors and non-survivors.

	**ePVS survivors**	**ePVS non-survivors**	***P*-value**
1 year	−3.68 (−10.92-4.22)	5.29 (−1.55-13.55)	0.005
Overall follow-up	−4.80 (10.00)	3.04 (12.23)	0.001
	**Duarte's PVS survivors**	**Duarte's PVS non-survivors**	***P*** **-value**
1 year	4.84 (4.19-6.77)	6.78 (5.44-7.83)	0.006
Overall follow-up	4.66 (4.17-6.13)	6.02 (4.80-7.72)	0.002

*Data is presented as mean ± standard deviation (SD) or median (interquartile range)*.

Survivors had a significantly lower ePVS after 1 year following the intervention (median ePVS −3.68 [IQR: −10.92-4.22] vs. 5.29 [IQR: −1.55-13.55], *p* = 0.005), and over the course of the overall follow-up (mean ePVS −4.80 ± 10.00 vs. 3.04 ± 12.23, *p* = 0.001).

Survivors had a significantly lower Duarte's PVS after 1 year following the intervention (median Duarte's PVS 4.84 [IQR: 4.19-6.77] vs. 6.78 [IQR: 5.44-7.83], *p* = 0.006), and over the course of the overall follow-up (median Duarte's PVS 4.66 [IQR: 4.17-6.13] vs. 6.02 [IQR: 4.80-7.72], *p* = 0.002).

There was a significant difference between both ePVS and Duarte's PVS in survivors and non-survivors at 1-year and during the overall follow-up period.

### Survival Stratified According to the ePVS and Duarte's PVS Cut-Off

**Figure 1:**
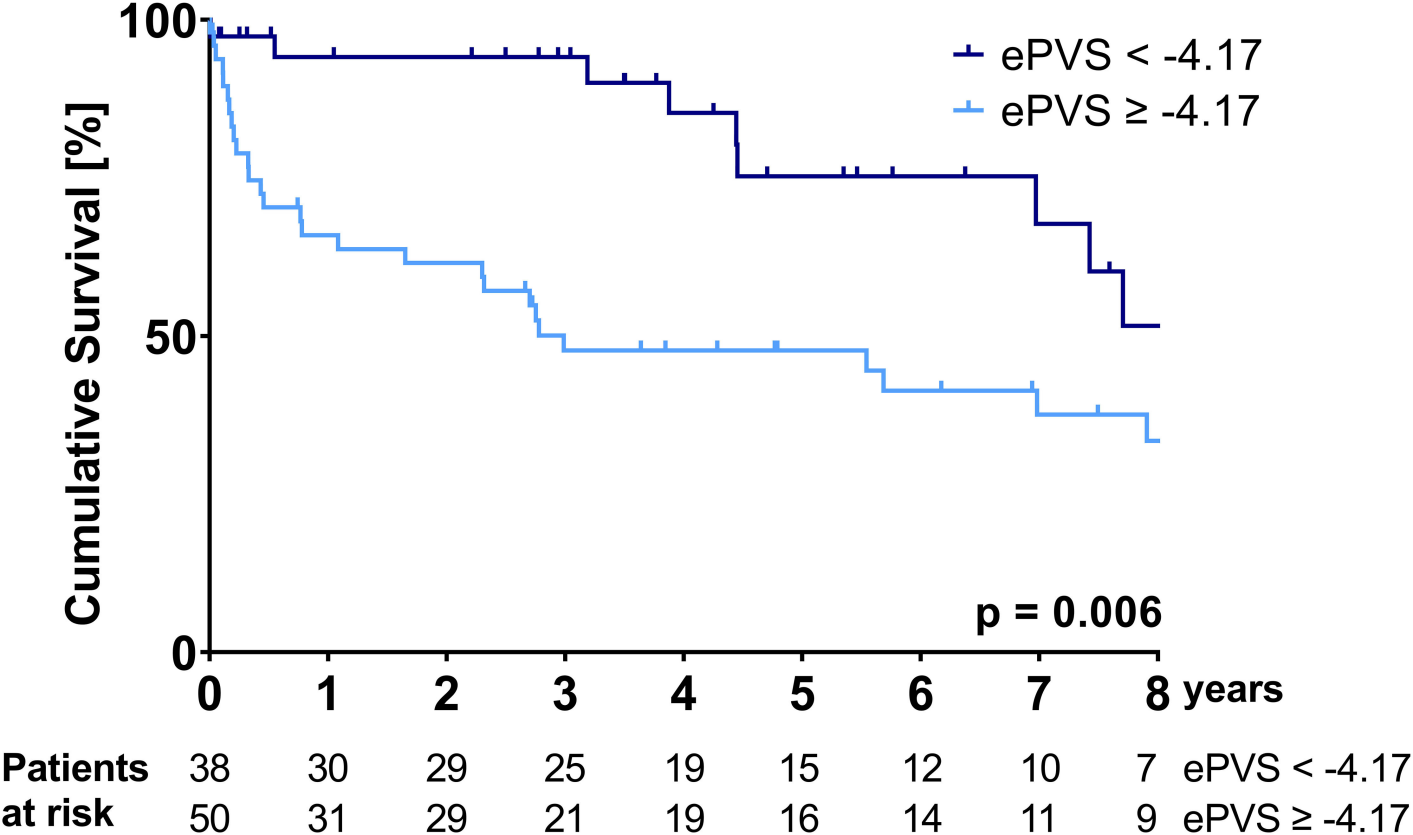
Kaplan-Meier survival curve over the course of the follow-up period—stratification according to the ePVS cut-off of −4.17.

**Figure 2:**
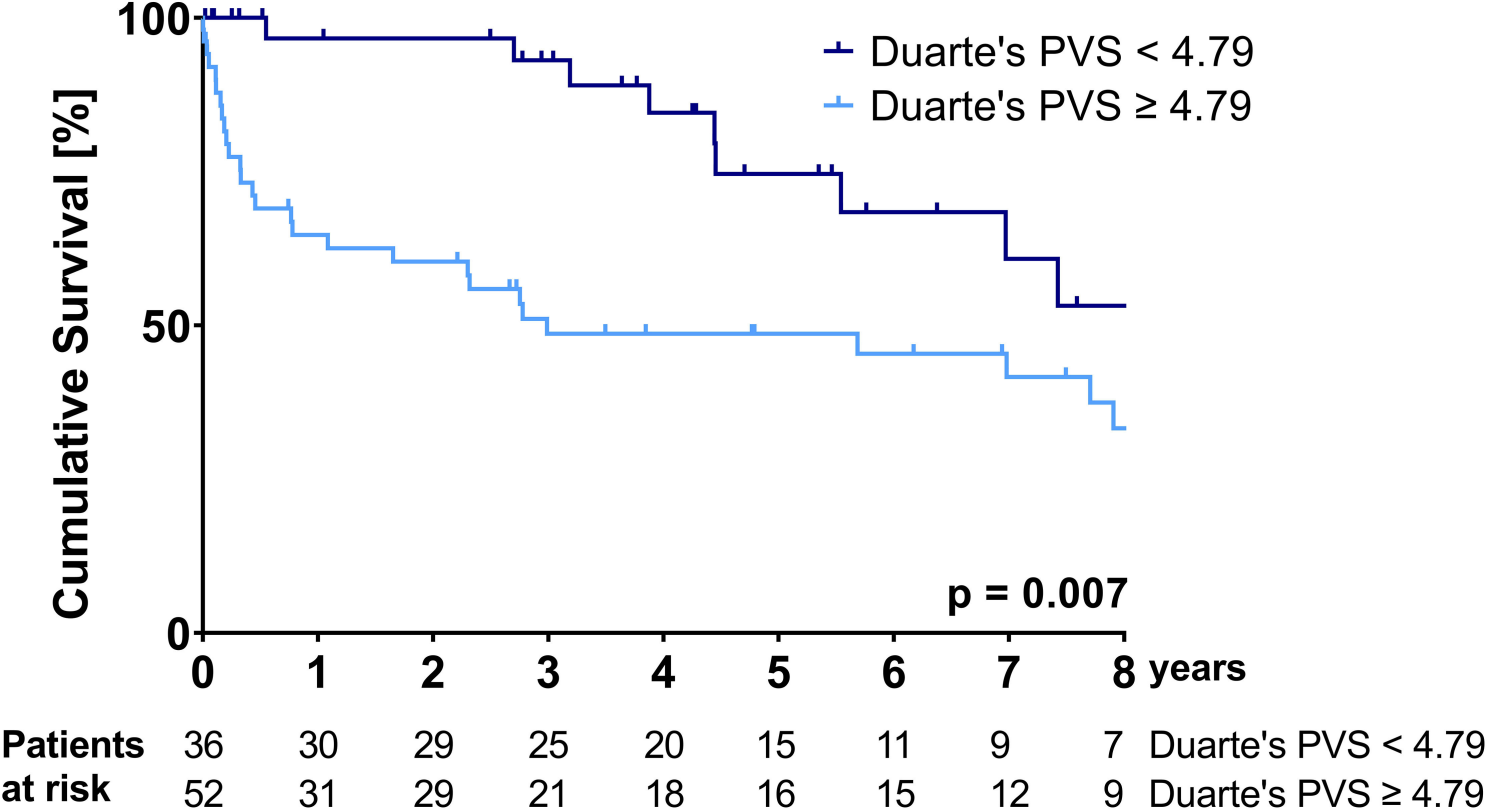
Kaplan-Meier survival curve over the course of the follow-up period—stratification according to the Duarte's PVS cut-off of 4.79.

Kaplan-Meier curves illustrating the survival of patients stratified according to the ePVS and Duarte's PVS cut-offs are shown in Figures 1, 2.

Patients with an ePVS of ≥-4.17 and patients with a Duarte's PVS of ≥4.79 had a significantly higher mortality over the follow-up period (median survival ePVS ≥ −4.17: 35.9 (95%CI: 0.0-80.7) months vs. median survival ePVS < – 4.17: not reached during follow-up, *p* = 0.006; median survival Duarte's PVS ≥ 4.79: 35.8 (95%CI: 0.0-92.7) months vs. median survival Duarte's PVS ≤ 4.79: not reached during follow-up, *p* = 0.007). The cumulative probability of survival at 1 and 3 years for the ePVS ≥ −4.17 group was 65.9 and 47.7%, respectively, compared to 94.1 and 94.1% for the ePVS < −4.17 group. The cumulative probability of survival at 1 and 3 years for the Duarte's PVS ≥ 4.79 group was 64.7 and 48.6%, respectively, compared to 96.7 and 93.1% for the Duarte's PVS <4.79 group.

### Predictors of Mortality

Detailed results of the univariate and multivariate analysis for predictors of mortality are shown in Table 4. Variables included in the multivariate analysis included isolated tricuspid valve replacement, EuroSCORE II, functional regurgitation, alkaline phosphatase levels, gamma-glutamyltransferase levels, CRP, as well as ePVS and Duarte's PVS. Of these variables, only ePVS and gamma-glutamyltransferase reached significance in the multivariate analysis (*p* = 0.04 and *p* = 0.027), whereas EuroSCORE II approached significance (*p* = 0.055).

**Table 4:** Predictors of mortality—univariate and multivariate analysis.

	**Univariate analysis**			**Multivariate analysis**		
**Variable name**	**Hazard ratio**	**95% confidence interval**	***P*-value**	**Adjusted hazard ratio**	**95% confidence interval**	***P*-value**
Age	1.026	1.006-1.046	0.010			
Baseline LVEF	0.966	0.940-0.993	0.014			
Elective surgery	0.519	0.275-0.980	0.043			
Urgent surgery	1.970	1.042-3.723	0.037			
Isolated TV repair	0.540	0.287-1.014	0.055			
Isolated TV replacement*	1.853	0.986-3.484	0.055			
EuroSCORE II*	1.054	1.018-1.092	0.003	1.039	0.999-1.079	0.055
NYHA class III/IV	2.667	1.035-6.872	0.042			
Diabetes mellitus	2.494	1.112-5.544	0.025			
Extracardiac arteriopathy	5.172	1.540-17.368	0.008			
Functional regurgitation*	0.561	0.282-1.113	0.098			
BMI	0.994	0.931-1.061	0.861			
Any previous intravenous drug use	1.129	0.541-2.354	0.746			
Active intravenous drug use	0.763	0.230-2.525	0.657			
Endocarditis related TR	1.078	0.552-2.102	0.827			
Any noncardiac chronic liver condition	1.805	0.936-3.480	0.078			
Active smoker	1.748	0.797-3.831	0.163			
Arterial hypertension	1.673	0.852-3.283	0.135			
Pacemaker/ICD	1.342	0.679-2.653	0.398			
COPD	1.343	0.690-2.613	0.385			
Chronic kidney disease	1.258	0.652-2.430	0.494			
Active endocarditis	0.966	0.462-2.091	0.927			
Pacemaker-related TR	1.382	0.576-3.320	0.469			
Redo surgery	0.790	0.359-1.403	0.323			
Previous surgical left heart valve intervention	0.894	0.391-2.044	0.791			
eGFR	0.990	0.979-1.001	0.074			
Hematocrit	0.935	0.889-0.984	0.010			
Hemoglobin	0.842	0.727-0.975	0.021			
Erythrocyte count	0.485	0.307-0.764	0.002			
proBNP	1.000	1.000-1.000	0.077			
Alkaline phosphatase*	1.005	1.002-1.008	0.001			
GGT*	1.003	1.000-1.006	0.026	1,004	1.001-1.007	0.004
CRP*	1.060	0.999-1.125	0.052			
Albumin	0.948	0.915-0.983	0.004			
Protein	0.981	0.959-1.003	0.094			
Sodium	0.940	0.883-1.001	0.052			
ePVS < −4.17*	0.360	0.170-0.765	0.008	0.431	0.188-0.905	0.027
Duarte's PVS <4.79*	0.352	0.161-0.771	0.009			
Platelet count	0.999	0.995-1.002	0.445			
Creatinine	0.975	0.603-1.579	0.919			
LDH	0.999	0.998-1.001	0.558			
Total bilirubin	1.060	0.899-1.250	0.490			

*Variables included in the multivariate Cox regression are indicated with an asterisk. LVEF, left ventricular ejection fraction; TV, tricuspid valve; NYHA, New York Heart Association; BMI, body mass index; ICD, implantable cardioverter-defibrillator; COPD, chronic obstructive pulmonary disease; eGFR, estimated glomerular filtration rate; BNP, brain natriuretic peptide; GGM, gamma-glutamyltransferase; CRP, C-reactive protein; LDH, lactate dehydrogenase*.

### Additional Considerations: NYHA Class and Renal Function

A significantly higher proportion of patients with ePVS ≥ −4.17 had NYHA class III/IV (83.7 vs. 64.9% in patients with ePVS < −4.17; *p* = 0.044). The median survival was significantly longer in patients with NYHA I/II (log-rank *p* = 0.035), with 1-year and 3-year survival probabilities of 83.6 and 78.0% in NYHA I/II as compared to 74.9 and 63.2% in NYHA III/IV. Following the sub-stratification according to ePVS levels, the individual contribution of both parameters in terms of median survival became clearer (log-rank *p* = 0.018), with a 1-year survival probability of 90.9% in ePVS < −4.17 + NYHAI/II, as compared to 64.1% in ePVS ≥ −4.17 + NYHAIII/IV.

In our study, eGFR ≥ 60 ml/min/1.73 m^2^ was not significantly associated with mortality (log-rank *p* = 0.790). Additionally, we found no significant correlation between ePVS and eGFR (r_s_: −0.108; *p* = 0.330) and no significant difference between ePVS levels in patients with eGFR ≥ 60 ml/min/1.73 m^2^ as compared to patients with eGFR <60 ml/min/1.73 m^2^.

## Discussion

This study provides a promising initial assessment of ePVS, an easily obtainable indicator of the plasma volume and degree of subclinical congestion, for the risk stratification of patients undergoing isolated tricuspid valve surgery.

Heart failure is highly prevalent in patients undergoing isolated tricuspid valve surgery (2). Methods for noninvasively assessing the severity of congestion and identifying high-risk patients are required. Our study found a significant difference in baseline ePVS between patients who survived during the first year of follow-up and those who did not. This finding also remained valid for the overall follow-up period. The ePVS cut-off we identified from our 1-year follow-up data was a significant predictor of the overall all-cause mortality in both the univariate and multivariate analyses. The other predictor of overall mortality in the multivariate model was the level of gamma-glutamyltransferase (GGT), whereas the EuroSCORE II approached statistical significance. In order to expand the analysis to a non-weight dependent calculation, we also performed a second set of analyses using PVS values calculated by the Duarte formula. There were significant differences between Duarte's PVS in survivors and non-survivors, and Duarte's PVS was significant in the univariate Cox regression, but not in the multivariate analysis.

Isolated tricuspid valve surgery is performed substantially less frequently than other valve interventions (6). Almost four fifths of all surgeries involving the tricuspid valve in adults are performed concomitantly with other procedures (36). As indicated by the low interventional volumes, and when compared to the overall prevalence of tricuspid valve disease, the indication for tricuspid valve surgery was and is often set too conservatively or too late (37). Several factors related to an impaired immediate and long-term survival have been identified. Dreyfus et al. identified NYHA class III/IV as a predictor of mid-term mortality (3). In accordance with these findings, the ePVS ≥ −4.17 cohort, which had a lower overall survival, had a significantly higher proportion of NYHA III/IV patients. In our patient cohort, individuals with a higher NYHA class (III/IV) had a significantly lower survival compared to patients with NYHA class I/II. However, a sub-stratification according to the ePVS cut-off and NYHA class, despite being statistically significant, resulted in small group sizes and crossing survival curves. Thus, these results must be interpreted with caution and validated by future studies.

Interestingly, more patients in the ePVS < −4.17 cohort, which had a higher overall survival, suffered from functional tricuspid regurgitation, which was also linked to a poorer survival in the study by Dreyfus et al. (3). Tricuspid valve replacement was performed less often than tricuspid valve repair in this cohort, and less frequently when compared to the ePVS ≥ −4.17 cohort. Additionally, fewer in-hospital deaths were observed in the ePVS < −4.17 cohort. Tricuspid valve replacement was shown to carry a higher mortality compared to tricuspid valve repair by Zack et al., although other analyses have delivered conflicting findings (6, 38). The ePVS < −4.17 cohort had fewer cases of endocarditis-related tricuspid regurgitation and intravenous drug use. Intravenous drug users are known to have an impaired long-term survival following isolated tricuspid endocarditis-related surgery and are prone to a higher recurrence of valve endocarditis (39, 40). In the ePVS < −4.17 cohort, significantly more elective surgeries and significantly fewer urgent interventions were performed. Thus, ePVS could represent a surrogate parameter for late referral or delayed surgery. Delayed or urgently performed surgery has been linked to a worse outcome in isolated tricuspid valve surgery, which is also mirrored by the two (5.2%) in-hospital deaths in the ePVS < −4.17 cohort compared to five deaths (10.0%) in the ePVS ≥ −4.17 cohort. However, this finding did not reach statistical significance, possibly due to the small overall incidence of this outcome. Notably, patients with an ePVS < −4.17 had lower levels of GGT, although not statistically significant. In the multivariate analysis, higher GGT levels were statistically significant predictors of overall mortality. A large population study has identified elevated GGT levels as independent predictors of all-cause and cardiovascular mortality (41). Elevated GGT levels have also been described in patients in the beginning stages of heart failure and linked to their NYHA class (42). The third predictor of a poorer outcome was a higher EuroSCORE II, which was lower in the ePVS < −4.17 cohort, but did not reach statistical significance in the multivariate model. Notably, the EuroSCORE II was primarily developed with operative risk assessment in mind. Nonetheless, due to the included clinical parameters, it inevitably correlates with later outcomes, as demonstrated by Wang et al. for isolated tricuspid valve surgery (43, 44).

Our study identified an ePVS of −4.17 as the optimal cut-off point for ePVS when assessing the 1-year mortality following isolated tricuspid valve surgery. The ePVS cut-off most notably agrees with a −4 cut-off identified in a large cohort study by Ling et al. published in 2015, which included over 5,000 patients with heart failure and was also validated in a smaller outpatient cohort (45). This study also included an additional validation step which compared the calculated ePVS to the values obtained via the current gold-standard nuclear medicine plasma volume measurement technique and discovered a satisfactory correlation between the measured and calculated values (45). Notably, the same cut-off was the basis for a study in 600 TAVI patients, where patients with an ePVS past this cut-off also demonstrated significantly worse postinterventional outcomes (46). Slightly lower ePVS cut-offs in the same range were proposed by Martens et al. and Seoudy et al. (23, 33). In the study conducted by Martens et al. on a large cohort of heart failure patients the measured plasma volume and calculated ePVS were found to be comparable (23). In their mixed cohort of patients with HFpEF, heart failure with a mid-range (HFmEF) and reduced ejection fraction (HFrEF), a −6.5 cut-off was identified as the optimal cut-off in terms of heart failure related hospitalization and all-cause mortality (23). Seoudy et al. identified an ePVS cut-off of −5.4 as a significant predictor of these outcomes in a TAVI cohort at 1 year after the intervention (33). Interestingly, Schaefer et al. identified a higher ePVS cut-off of 3.1 when analyzing the correlation of the calculated plasma volume status with short- and long-term outcomes in patients undergoing mitral valve surgery (34). In summary, the ePVS cut-off identified by our study is in agreement with data from other trials in different patient collectives and thus provides initial proof that, on the one hand, ePVS might represent a viable stratification parameter for patients undergoing isolated tricuspid valve surgery, and on the other hand, ePVS might represent an overarching heart failure-related stratification parameter that might play a key role in identifying at-risk cardiac surgery patients undergoing a number of different interventions.

Within our study, we also assessed a different approach to calculating the plasma volume from the hematocrit and hemoglobin values at baseline, as previously described by Duarte et al. (20). For Duarte's PVS, 4.79 was the optimal cut-off in our study. Notably, this cut-off is similar to the one identified by Lin et al. in patients with systolic heart failure (47). In their study, a Duarte's PVS higher than 4.35 was associated with more frequent hospitalization and a higher overall mortality (47). In a large cohort of patients admitted to the emergency department with dyspnea, Duarte's PVS higher than 4.17 and 5.12 in particular was linked to significantly worse in-hospital survival, whereas Duarte's PVS > 5.12 increased the likelihood for the diagnosis of acute heart failure later on (48). Thus, the cut-off identified within our study lies well within the range of values identified by other studies.

In our study, ePVS calculated at baseline was a significant predictor of mortality. A study by Tamaki et al. evaluated the prognostic value of plasma volume calculations performed by all three aforementioned formulas: the Hakim, Strauss and Duarte formula for the prediction of outcomes of patients admitted for acutely decompensated heart failure following discharge (28). In their analysis, only ePVS calculated using the Hakim formula at baseline and before discharge reliably predicted the primary outcome (28). In a study by Kobayashi et al., Duarte's PVS measured at discharge was a reliable predictor of outcomes in patients admitted for acute decompensated heart failure, whereas the admission values and the overall change during the stay were not (24). This highlights the need for studies involving plasma volume status assessments during multiple timepoints to determine not only the optimal cut-off, but also the optimal timepoint for plasma volume calculations for patient risk stratification.

Despite the promising results, our study has a few limitations inherent to its retrospective character and small sample size. Most importantly, numerical echocardiography data were not electronically recorded for a large part of our patient collective, thus precluding us from conducting detailed analyses in this regard. Furthermore, the small sample size limited the combined sub-stratification analysis according to other key parameters, such as NYHA class.

In conclusion, ePVS is an easily obtainable risk score for patients undergoing isolated tricuspid valve surgery capable of predicting mid- and long-term outcomes after isolated tricuspid valve surgery. Our study proposes an ePVS cut-off of −4.17 for long-term risk stratification and a 4.79 cut-off for PVS calculated using Duarte's formula.

## Data Availability Statement

The raw data supporting the conclusions of this article will be made available by the corresponding author, without undue reservation.

## Ethics Statement

The studies involving human participants were reviewed and approved by Ethics Committee of the Medical University of Vienna, Vienna, Austria. Written informed consent for participation was not required for this study in accordance with the national legislation and the institutional requirements.

## Author Contributions

MM, MR, and MA: conceptualization. MM and EH: methodology. BH, EH, and MM: formal analysis. GL, AK, DW, and MA: resources. MR and EH: data curation. EH and MM: writing—original draft preparation. PW, IC, MR, GL, AK, DW, and MA: writing—review and editing. EH and BH: visualization. MM: supervision. MR: project administration. All authors have read and agreed to the published version of the manuscript.

## Conflict of Interest

MM received institutional grants, research support, speaker honoraria, and travel compensation from Edwards Lifesciences, Symetis SA, Jena Valve, Boston Scientific, Medtronic, Abbott and Novartis. MA is a proctor for Edwards Lifesciences and Abbott Laboratories and an advisor to Medtronic. DW is a proctor for Abbott and a scientific advisor for Fresenius/Xenios. The remaining authors declare that the research was conducted in the absence of any commercial or financial relationships that could be construed as a potential conflict of interest.

## Publisher's Note

All claims expressed in this article are solely those of the authors and do not necessarily represent those of their affiliated organizations, or those of the publisher, the editors and the reviewers. Any product that may be evaluated in this article, or claim that may be made by its manufacturer, is not guaranteed or endorsed by the publisher.
